# Influences of prenatal and postnatal maternal depression on amygdala volume and microstructure in young children

**DOI:** 10.1038/tp.2017.74

**Published:** 2017-04-25

**Authors:** D J Wen, J S Poh, S N Ni, Y-S Chong, H Chen, K Kwek, L P Shek, P D Gluckman, M V Fortier, M J Meaney, A Qiu

**Affiliations:** 1 Department of Biomedical Engineering, Clinical Imaging Research Center, National University of Singapore, Singapore, Singapore; 2 Singapore Institute for Clinical Sciences, Singapore, Singapore; 3 Department of Obstetrics and Gynaecology, Yong Loo Lin School of Medicine, National University of Singapore, National University Health System, Singapore, Singapore; 4 KK Women's and Children's Hospital, Singapore, Singapore; 5 Department of Pediatrics, Khoo Teck Puat – National University Children's Medical Institute, National University of Singapore, Singapore, Singapore; 6 Department of Diagnostic and Interventional Imaging, KK Women's and Children's Hospital, Singapore, Singapore; 7 Ludmer Centre for Neuroinformatics and Mental Health, Douglas Mental Health University Institute, McGill University, Montreal, QC, Canada; 8 Sackler Program for Epigenetics and Psychobiology at McGill University, Montreal, QC, Canada

## Abstract

Maternal depressive symptoms influence neurodevelopment in the offspring. Such effects may appear to be gender-dependent. The present study examined contributions of prenatal and postnatal maternal depressive symptoms to the volume and microstructure of the amygdala in 4.5-year-old boys and girls. Prenatal maternal depressive symptoms were measured using the Edinburgh Postnatal Depression Scale (EPDS) at 26 weeks of gestation. Postnatal maternal depression was assessed at 3 months using the EPDS and at 1, 2, 3 and 4.5 years using the Beck's Depression Inventory-II. Structural magnetic resonance imaging and diffusion tensor imaging were performed with 4.5-year-old children to extract the volume and fractional anisotropy (FA) values of the amygdala. Our results showed that greater prenatal maternal depressive symptoms were associated with larger right amygdala volume in girls, but not in boys. Increased postnatal maternal depressive symptoms were associated with higher right amygdala FA in the overall sample and girls, but not in boys. These results support the role of variation in right amygdala structure in transmission of maternal depression to the offspring, particularly to girls. The differential effects of prenatal and postnatal maternal depressive symptoms on the volume and FA of the right amygdala suggest the importance of the timing of exposure to maternal depressive symptoms in brain development of girls. This further underscores the need for intervention targeting both prenatal and postnatal maternal depression to girls in preventing adverse child outcomes.

## Introduction

Perinatal maternal depression is associated with an increased risk for emotional,^1, 2, 3^ behavioral^4^ and cognitive problems,^5^ as well as multiple forms of psychopathology^6, 7, 8^ in the offspring. Compelling evidence also suggests the influence of perinatal maternal depressive symptoms on brain development,^9, 10, 11, 12, 13, 14, 15^ particularly on the amygdala, a brain structure critical for emotional processing,^16, 17^ stress reactivity^18^ and vulnerability to depression.^19^ Recent research showed a significant association between prenatal maternal depressive symptoms and the amygdala microstructure in neonates shortly after birth.^11^ Prenatal maternal depressive symptoms also modulate the development of amygdala functional organization in the first 6 months of life.^12^ Beyond prenatal maternal depression, increased postnatal maternal depressive symptoms associate with a larger amygdala volume in 10-year-old children.^10^ These are in line with findings on an increased amygdala volume in children under institutional rearing conditions.^20, 21^ Likewise, the first-degree relatives of patients with major depressive disorder were found to have a larger amygdala volume than healthy controls.^22^ These findings highlight the vulnerability of amygdala development in offspring to the exposure of an early adverse environment. Hence, the amygdala can be an interesting candidate brain structure for understanding the biological basis of the association between maternal emotional well-being and the mental health of the offspring. Nevertheless, the timing for the influence of maternal depression on amygdala development in early childhood remains unclear, which complicates models of risk as well as the design and timing of preventive interventions.

Prenatal and postnatal maternal depressive symptoms have an impact on the offspring possibly through distinct pathways. Depressed mothers during pregnancy exhibit a number of physiological changes that may influence intrauterine environment and hence implicate alterations in fetal development. In contrast, postnatal maternal depression influences the offspring most likely through forms of parenting^14, 23, 24^ that enhance fearfulness, social withdrawal^25, 26, 27, 28^ and predict an increased risk for later psychopathology in the offspring.^29^ One would expect that prenatal and postnatal maternal depressive symptoms independently influence brain development in offspring. Indeed, prenatal and postnatal maternal depression independently predicts an increased risk for depression in offspring.^30^ Interestingly, a very recent neuroimaging study suggested differential and independent influences of prenatal and postnatal maternal depressive symptoms on cortical morphology and white matter microstructure in children aged between 2.6 and 5.1 years old.^31^ Alternatively, environmental conditions at one stage in development influence the sensitivity to later conditions.^32^ Changes in the intrauterine environment due to prenatal maternal depression may increase the child's susceptibility to postnatal maternal depression. Although this hypothesis is less investigated, Lusby *et al.*^33^ recently showed that higher levels of postnatal depressive symptoms were associated with greater relative right frontal electroencephalogram asymmetry specifically among infants whose mothers had higher levels of prenatal depressive symptoms, suggesting an interaction effect of prenatal and postnatal maternal depressive symptoms on neurodevelopment. Nevertheless, there is a lack of knowledge on how prenatal and postnatal maternal depressive symptoms interplay and impact brain development in offspring, particularly the amygdala.

An extensive meta-analysis^34^ shows that in community samples, the association between maternal depressive symptoms and internalizing problems is stronger in girls. Likewise, the association between maternal emotional well-being and amygdala-prefrontal connectivity is unique to girls.^35^ A higher maternal cortisol level in early gestation is associated with larger right amygdala volume in childhood, but only in girls.^9^ These findings underscore the importance of gender-dependent developmental pathways of neuronal substrates that underlie the risk for transgenerational transmission of depression from mother to child.

In this study, we examined the extent to which prenatal and postnatal maternal depressive symptoms interactively or independently contribute to the development of the amygdala structure in 4.5-year-old children. We employed both structural magnetic resonance imaging (MRI) and diffusion tensor imaging (DTI)^36^ to assess the volume and microstructure of the amygdala. Prenatal and postnatal maternal depressive symptoms were assessed at multiple time points. Given considerable evidence for sexually dimorphic associations between maternal mental health and neurodevelopmental outcomes,^37, 38, 39, 40, 41, 42, 43, 44, 45, 46, 47^ we further explored the above questions in the full, female and male samples, respectively, to elucidate potential gender differences of the amygdala volume and microstructure in response to maternal depressive symptoms in early life. If both prenatal and postnatal maternal depressive symptoms interactively influence amygdala development, we hypothesized that this interaction would influence both the size and structural organization of the amygdala. If prenatal and postnatal maternal depressive symptoms independently contribute to amygdala development, the effects of maternal depressive symptoms on the size and structural organization may presumably be dependent on the underlying developmental processes of the brain, such as neurogenesis, synaptogenesis and so on. The pronounced relationship between maternal depressive symptoms and the amygdala would be greater in girls than in boys.

## Materials and methods

### Participants

Three hundred and forty-two mother–child dyads who participated in the prospective GUSTO (Growing Up in Singapore Towards healthy Outcomes) birth cohort study were recruited for neuroimaging study when the children were 4.5 years of age. The GUSTO cohort recruited pregnant Singapore citizens, or permanent residents of Chinese, Malay or Indian ethnic backgrounds from two major birthing hospitals in Singapore at the first antenatal visit (see Soh *et al.*^48^ for further details). The GUSTO study was approved by the National Healthcare Group Domain Specific Review Board and the Sing Health Centralized Institutional Review Board. Written informed consent was obtained from mothers.

Maternal education, ethnicity, age and monthly household income were extracted from survey questionnaires conducted as part of a scheduled appointment during the 26th week of pregnancy. Birth outcomes, including gestational age, birth weight, APGAR (Appearance, Pulse, Grimace, Activity, and Respiration) score and gender, were obtained from the hospital record.

This study only included children with gestational age ⩾34 weeks, birth weight ⩾2 kg and a 5-min APGAR score ⩾8 to avoid potential effects of birth complications on the brain development and with maternal reports on depression scales.

### Maternal depression scales

The Edinburgh Postnatal Depression Scale (EPDS) questionnaire was administered to mothers at 26 weeks of pregnancy and 3 months after delivery to assess depressive symptomatology. The EPDS^49^ is a widely used 10-item self-report scale designed as a screening instrument for postnatal depression and valid for use in prenatal and early postnatal depression. Each item of the EPDS is scored on a four-point scale (0–3), and items 3 and 5–10 are reverse-scored.

The Beck's Depression Inventory-II (BDI-II) was administered to mothers at 1, 2, 3 and 4.5 years postpartum. The BDI-II is a widely used 21-item questionnaire that assesses the existence and severity of symptoms of depression and predicts the severity of clinical depressive symptoms.^50^ Each item of the BDI-II is scored on a four-point scale (0–3). Higher total scores indicate more severe depressive symptoms.

Prorating imputation was performed when 8 or 9 questions were answered on the EPDS or 19 or 20 questions were answered for the BDI-II. All EPDS and BDI measures were standardized. As the scores of postnatal maternal depressive symptoms were highly correlated with each other (*r*>0.5, *P*<0.001), the averaged score across the postnatal points was computed to quantify the severity of postnatal maternal depressive symptoms up to 4.5 years postpartum.

### MRI acquisition and quality check

Children underwent MRI scans at age of 4.5 years (±1month) using a 3 T Siemens Skyra scanner (Siemens, Munich, Germany) with a 32-channel head coil at KK Women's and Children's Hospital. Children were recruited during a 4-year home visit. Children went through an MRI home training program prior to the MRI visit and on-site MRI training (see details in the Supplementary Material). The image protocols were: (i) high-resolution isotropic T_1_-weighted magnetization prepared rapid gradient recalled echo (192 slices, 1 mm thickness, in-plane resolution 1 mm, sagittal acquisition, field of view 192 × 192 mm, matrix=192 × 192, repetition time=2000 ms, echo time=2.08 ms, inversion time=877 ms, flip angle=9°, scanning time=3.5 min); (ii) isotropic axial diffusion-weighted imaging protocol (single-shot echo-planar sequence, 69 slices of 2.0 mm thickness, with no inter-slice gaps, matrix 96 × 96, field of view 192 × 192 mm, repetition time=8200 ms, echo time=85 ms, flip angle=90°, 30 diffusion-weighted images with *b*=1000 s mm^−2^, 5 baseline images without diffusion weighting, GRAPPA=3, scanning time=5.5 min).

The image quality was verified immediately after the acquisition through visual inspection when children were still in the scanner. A scan was repeated when ring artifact on T_1_-weighted images and signal loss on DTI were large (see an example in Supplementary Figure S1). The image was removed from the study if no acceptable image was acquired after three repetitions.

### Structural MRI and DTI analysis

FreeSurfer was used to label each voxel in the T_1_-weighted image as gray matter, or white matter, or cerebrospinal fluid, or subcortical structures (for example, hippocampus, amygdala, thalamus, caudate, putamen and globus pallidus).^51^ FreeSurfer employed a Markov random field model that requests for a prior probability obtained from a training data set with T_1_-weighted images and their manual structural labels. In this study, we reconstructed the prior probability in the Markov random field model based on the manual segmentation of 30 Asian children and embedded it in FreeSurfer (replacing RB_all_2008-03-26.gca under freesurfer/average). FreeSurfer was then performed to each T_1_-weighted image in this study. Post-processing quality check was conducted following by the instruction on https://surfer.nmr.mgh.harvard.edu/fswiki/FsTutorial/TroubleshootingData. The segmentation accuracy was assessed using a volume overlap ratio (VOR) between the automated and manual segmentation.^51^ The VOR values for the amygdala is 0.90±0.05, suggesting the high accuracy of the automated segmentation when compared with the manual labeling.

Within individual subjects, diffusion-weighted images were corrected for motion and eddy current distortions using affine transformation to the image without diffusion weighting.^52^ Using multivariate least-square fitting, six elements of the diffusion tensor were then determined, from which fractional anisotropy (FA) was calculated. The amygdala mask in the T_1_-weighted image was then superimposed to the FA images through affine transformation obtained between the image without diffusion weighting and T_1_-weighted image. Mean FA values were computed for the amygdala and used in the following statistical analysis.

### Statistical analysis

Multiple regression analyses were used to examine the interactive, independent and main effects of prenatal and postnatal maternal depression on the amygdala volume and FA. These regression analyses were repeated for three groups of subjects: (1) the overall sample, (2) girls and (3) boys.

#### Interaction model (interactive effects)

The interaction of prenatal and postnatal maternal depression was formed as the product of the two standardized predictors. A hierarchical order of entry was used to enter predictors. Covariates were entered in the first block followed by prenatal maternal depression and postnatal maternal depression in the second block and the interaction term in the third block.

#### Reduced model (independent effects)

In cases where the interaction term was not significant, a reduced regression model without the inclusion of the interaction term was used to consider independent effects of prenatal and postnatal maternal depression on the amygdala volume and FA.

#### Separate models (main effects)

The main effects of prenatal or postnatal maternal depression on bilateral amygdala volume and FA were also examined in two separate models. In the first model, covariates were entered in the first block followed by prenatal maternal depression in the second block. In the second model, covariates were entered in the first block followed by postnatal maternal depression in the second block.

### Confounding variables

This study considered variables that are either related to the amygdala volume or risk factors for maternal depression. Hence, the age at MRI, maternal ethnicity, maternal education and total brain volume were included as common covariates in the regression analysis to control for potential influences on the amygdala volume. The same covariates except for total brain volume were included in the regression analysis on the amygdala FA. In addition, gender was also used as covariate when analyzing the overall sample. Covariates that had a categorical level of measurement (that is, maternal ethnicity) were dummy coded before they were entered into the regression model to ensure their suitability for regression. As household income was highly correlated with maternal education (*r*=0.521; *P*<0.001) and maternal age was highly correlated with household income (*r*=0.281; *P*<0.001), these were not further included as covariates to avoid potential collinearity problems.

## Results

### Demographics

Of the 342 subjects who underwent MRI, 77 subjects had unusable T1 or DTI data due to image quality, 4 did not meet the inclusion criteria and 26 mothers of infants did not complete depression questionnaires (that is, EPDS or BDI). Hence, the total sample size in this study included 235 subjects. Among them, 203 (95 boys and 108 girls) had good T1 data and 188 (88 boys and 100 girls) had good DTI data. Table 1 lists the demographic information of the full, boy and girl samples that were used in this study. We also list the demographic information of 77 subjects who were not used in this study due to image quality in Supplementary Table S1.

The mothers of boys and girls did not differ in prenatal (*t*_233_=1.68, *P*=0.094), and postnatal maternal depressive symptoms (*t*_233_=1.12, *P*=0.264), maternal education (*t*_231_=−0.650, *P*=0.516) and maternal ethnicity (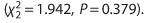
=1.942, *P*=0.379).

### Relations between maternal depressive symptoms and the amygdala volume

The full sample (Table 2) showed no interaction of prenatal and postnatal maternal depressive symptoms on the left (*β*=0.005, *P*=0.944, df=192) or right (*β*=0.098, *P*=0.141, df=191) amygdala volumes. Similarly, there was no independent effect of prenatal maternal depressive symptoms on left (*β*=0.095, *P*=0.150, df=193) and right (*β*=0.097, *P*=0.137, df=192) amygdala volumes after adjusting for postnatal maternal depressive symptoms. There were no effects of postnatal maternal depressive symptoms on left (*β*=0.030, *P*=0.648, df=193) or right (*β*=−0.003, *P*=0.958, df=192) amygdala volumes after adjusting for prenatal maternal depressive symptoms.

The main effects remained largely similar when prenatal and postnatal maternal depressive symptoms were considered in separate models. There was a trend for a significant effect for prenatal maternal depressive symptoms in relation with the left amygdala volume (*β*=0.109, *P*=0.061, df=194) and right amygdala volume (*β*=0.096, *P*=0.097, df=193). Postnatal maternal depressive symptoms did not significantly predict left (*β*=0.074, *P*=0.203, df=194) or right (*β*=0.042, *P*=0.466, df=193) amygdala volume.

**Figure 1:**
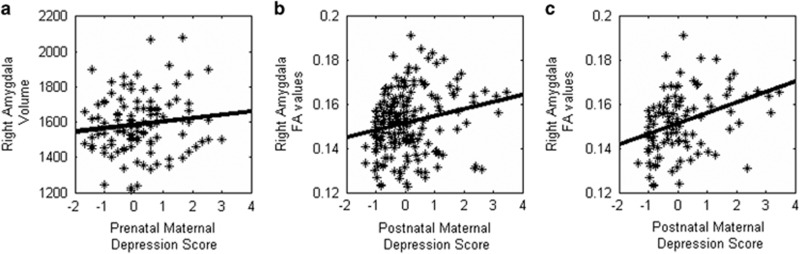
Scatter plots of (**a**) prenatal maternal depression score with right amygdala volume in the girls sample, (**b**) postnatal maternal depression score with right amygdala fractional anisotropy (FA) in the full sample and (**c**) postnatal maternal depression score with right amygdala FA in the girls sample.

The effects of prenatal and postnatal maternal depressive symptoms on the amygdala volumes in the boy sample were the same as those for the full sample (Table 2). Interestingly, in girls, greater prenatal maternal depressive symptoms predicted a larger right amygdala volume with (*β*=0.219, *P*=0.043, df=99; Table 2; Figure 1a) and without (*β*=0.195, *P*=0.042, df=100) adjusting for postnatal maternal depressive symptoms. There were no independent effects of postnatal maternal depressive symptoms on the left (*β*=0.009, *P*=0.930, df=99) or right (*β*=−0.050, *P*=0.622, df=99) amygdala volumes.

### Relations between maternal depressive symptoms and the amygdala microstructure

The full sample (Table 3) did not show a significant interaction of prenatal and postnatal maternal depressive symptoms on the left amygdala (*β*=−0.025, *P*=0.779, df=178) or right amygdala (*β*=−0.046, *P*=0.597, df=177) FA. There were no independent effects of prenatal maternal depressive symptoms on the left (*β*=0.026, *P*=0.768, df=179) or right (*β*=−0.020, *P*=0.811, df=178) amygdala FA after adjusting for postnatal maternal depressive symptoms. Likewise, there was no independent effect of postnatal maternal depression on left (*β*=0.105, *P*=0.218, df=179) amygdala FA. However, greater postnatal maternal depressive symptoms strongly predicted greater right (*β*=0.233, *P*=0.005, df=178) amygdala FA in the overall sample (Figure 1b).

The aforementioned relations remained essentially the same when prenatal and postnatal maternal depressive symptoms were entered into separate regression models. Prenatal maternal depressive symptoms did not predict the left amygdala (*β*=0.079, *P*=0.297, df=180) FA or right amygdala (*β*=0.097, *P*=0.195, df=179) FA. Postnatal maternal depressive symptoms did not significantly predict the left (*β*=0.117, *P*=0.112, df=180), but strongly significantly predicted the right (*β*=0.223, *P*=0.002, df=179) amygdala FA.

The effects of prenatal and postnatal maternal depressive symptoms on the amygdala microstructure in girls (Table 3; Figure 1c) were the same as those in the full sample. Again, in girls, greater postnatal maternal depressive symptoms predicted a higher right amygdala FA value with (*β*=0.348, *P*=0.001, df=92; Figure 1c) and without adjusting for prenatal maternal depressive symptoms (*β*=0.325, *P*=0.001, df=93). In contrast, the boy sample did not show any significant effects of prenatal and postnatal maternal depressive symptoms on the amygdala microstructure (Table 3).

## Discussion

The present study investigated the relationship of prenatal and postnatal maternal depressive symptoms with amygdala volume and microstructure in children using a community sample. This study did not show any evidence on interaction of prenatal and postnatal maternal depressive symptoms on amygdala structure. However, this study revealed that greater prenatal maternal depressive symptoms predicted a larger right amygdala volume in girls, whereas postnatal maternal depressive symptoms associated with right amygdala microstructure in the overall sample and in girls, but not in boys. These findings suggested independent, differential influences of prenatal and postnatal maternal depressive symptoms on the structural development of the amygdala, with evidence for gender-specific effects.

Our study underscored the importance of the right amygdala in relation to maternal depressive symptoms. Our findings are consistent with previous reports showing differential effects of maternal mood on the right versus the left amygdala.^9, 11^ Although the biological basis for such asymmetric development effects is unknown, our findings are consistent with the hypothesis that maternal emotional well-being selectively affects neural structures implicated in the processing of negatively valenced emotional information and in the accompanying of stress responses.^53, 54, 55, 56, 57^ This is particularly applied to the right amygdala because its activation associates with a negative attentional bias and is considered as an endophenotype for both anxiety and depression.^58^ During stress induction, the right amygdala responds at equally high levels for threat-related and positively valenced stimuli.^59^ Childhood maltreatment associates with both a selective increase in right amygdala volume^60^ and a negative attentional bias.^61^ Moreover, greater prenatal maternal depressive symptoms associate with the right amygdala microstructure in neonates shortly after birth^11^ and greater postnatal maternal depressive symptoms predict a larger right amygdala volume in 10-year-old children.^10^ These findings implicated that the right amygdala could be a neural origin for transgenerational transmission of risk for mood disorders from mother to child.

Though limited, there is evidence suggesting that children born to depressed mothers may be more susceptible to postnatal maternal depression.^1^ Greater levels of postnatal depressive symptoms associate with greater abnormal right frontal electroencephalogram asymmetry particularly among infants whose mothers had greater levels of depressive symptoms during pregnancy.^33^ This is contradictory to what was observed in this study on the right amygdala. This could be partially due to differences in the level of the severity of maternal depressive symptoms. This study was based on the community sample, whereas existing literature focuses on clinical samples of depressed mother–child dyads.^33^ On the other hand, these findings could suggest that interactive or independent effects of maternal depressive symptoms are brain region specific, which could be mediated via common or distinct mechanisms.

Prenatal and postnatal maternal depressive symptoms independently contributed to the amygdala structural development in 4.5-year-old girls, which is consistent with the findings on the cortisol level among 4.5-year-old children.^62^ Greater maternal depressive symptoms during pregnancy predicted larger right amygdala volume in 4.5-year-old girls. A greater severity of postnatal maternal depressive symptoms associated with higher right amygdala FA at 4.5 years of age, again with effects apparent in girls. On the other hand, Lupien *et al.*^10^ found that larger bilateral amygdala volumes in 10-year-old children continually exposed to postnatal maternal depression since birth compared to those without exposure of postnatal maternal depression. Thus, the influence of maternal depressive symptoms on the specific measure of the amygdala differs as a function of the age of the offspring. Indeed, using the same birth cohort, we previously found that greater prenatal maternal depressive symptoms associated with lower FA values of the right amygdala, but with no association with the amygdala volume in neonates shortly after birth.^11^ Even though biological mechanisms underlying these findings are unclear, these may not surprising given the fact that various ontogenetic processes of development occur in the brain at different periods of time,^63, 64^ such that structural imaging studies are tracking a ‘moving target'. Our findings are also consistent with the results of comparable studies of corticolimbic development showing that the nature of the environmental influence on neural structure is dependent upon developmental timing.^65^ For instance, the preschool age is a sensitive period for the influence of maternal support on the trajectory of hippocampal development.^66^ High maternal support was also found to be associated with lower activation of the amygdala to fearful faces in adolescence.^67^ Likewise, our studies provided new evidence that the influence of maternal depressive symptoms on the amygdala depends on developmental timing in early life.^11^ The development of the amygdala begins at an early embryonic stage^68^ and continues well into postnatal life.^69, 70^ Axon myelination is thought to peak during the early postnatal period,^71^ whereas the development of axonal connections is known to be dependent on the postnatal environment. The postnatal environment includes the influence of parental care, which associates with developmental trajectories in corticolimbic structures.^66^ The development of individual differences in the amygdala structural and functional connectivity is influenced by the quality of the postnatal rearing environment,^10, 20, 21, 72^ with evidence for accelerated maturation in response to environmental adversity.^73^

Our findings were apparent in 4.5-year-old girls not boys, suggesting that the maternal influences on the amygdala development may be dependent on gender, whereby the same environmental condition leads to differential effects on boys and girls. The association between maternal depressive symptoms and internalizing problems is stronger in girls.^34^ In line with these findings, prenatal stress also leads to sexually dimorphic developmental consequences later in life.^38, 39, 40, 41, 74^ There exist gender-specific responses of the fetal-placental unit to stress, where female foetuses are more susceptible to changes in stress levels.^75, 76^ There is similar evidence for a positive association between prenatal maternal cortisol levels and right amygdala volume in girls, but not boys at 7 years of age.^9^ Hence, gender-dependent effects on neurodevelopmental outcomes are not unique to the influence of maternal mood but they cut across various forms of maternal adversity, including maternal depression, maternal stress, maternal cortisol and so on. However, little is known on what mechanisms could explain such gender-dependent effects on neurodevelopmental outcomes. Although substantial evidence supports the idea that gender differences in response to stress may be partly due to sex hormones,^77, 78^ we reported that sexually dimorphic developmental consequences (for example, amygdala) due to prenatal maternal depression are not shown shortly after birth^11^ but become pronounced at age of 4.5 years using the same sample as that in this study. This raises the question on when sex hormones starts to play a role in differentiating developmental consequences due to maternal environment between boys and girls, which needs future investigation.

To the best of our knowledge, this study is the first to combine multimodal magnetic resonance images in probing amygdala volume and microstructure within a relatively large sample of young children with the objective of understanding the timing of the influences of maternal depressive symptoms on amygdala development. Moreover, several unique features of the study include the analysis of the interactive or independent effects of prenatal and postnatal maternal depressive symptoms on amygdala volume and FA as well as the assessment of gender effects in the sample. Nevertheless, in consideration of both scientific importance and subject burden, our study only assessed prenatal maternal depression at one time point, namely, 26 weeks of gestation, given the fact that the second and third trimesters during pregnancy are critical periods of neural migration and synaptogenesis in the fetal brain. Additional measurements would have allowed for a better understanding of the specificity of timing during pregnancy. However, the existing data suggest that individual differences in depressive symptoms are in general stable across pregnancy.^79, 80^ In addition, our assessment of maternal depression was based on a common screening tool designed to elicit a subjective report of emotional well-being, but did not constitute a clinical assessment. The reported results are thus best considered as being associated with self-reported depressive symptoms and not with clinical depression, *per se*. Furthermore, potential factors, such as parenting, should be considered to understand possible pathways mediating the association of maternal depression and the amygdala structure in children. Finally, imaging children at this age is very challenging. Our study discarded 22.5% images acquired because of signal loss that was inspected qualitatively rather than quantitatively due to the lack of ground truth. In general, training children outside the scanner can significantly improve image acquisition.^81^ Our study had carefully designed the training program to get children familiar and cooperative with the scanning procedure and instruction, and to improve image quality (Supplementary Material).

Our study sought to investigate the effect of prenatal and postnatal maternal depressive symptoms on the volume and microstructure of the amygdala in 4.5-year-old children and the presence of sexually dimorphic development. Our findings indicate the independent and differential contributions of prenatal and postnatal maternal depressive symptoms to amygdala volume and microstructure in girls, but not in boys. Our study thus emphasizes the importance of the timing of exposure to maternal depressive symptoms, which further facilitates the understanding of the plasticity and vulnerability of the brain to maternal depression in early life, as well as the importance of gender.

## Figures and Tables

**Table 1:** Demographics

*Measure*	*Overall sample (*N*=235)*	*Male sample (*N*=113)*	*Female sample (*N*=122)*
*Child characteristics*			
Gestational age (week), mean (s.d.)	38.75 (1.20)	38.56 (1.31)	38.93 (1.07)
Birth weight (g), mean (s.d.)	3111.69 (412.38)	3161.86 (431.93)	3065.23 (389.43)
APGAR score, mean (s.d.)	9.00 (0.07)	9.00 (0.00)	8.99 (0.09)
Age (year), mean (s.d.)	4.58 (0.08)	4.59 (0.09)	4.58 (0.07)
Total brain volume (cm^3^), mean (s.d.)	1212.61 (107.40)	1268.25 (98.11)	1161.04 (88.49)
Right amygdala volume (mm^3^), mean (s.d.)	1659.60 (197.98)	1741.61 (196.86)	1584.36 (167.41)
Left amygdala volume (mm^3^), mean (s.d.)	1583.81 (193.57)	1653.60 (199.22)	1519.14 (164.27)
Right amygdala FA, mean (s.d.)	0.15 (0.02)	0.15 (0.02)	0.15 (0.02)
Left amygdala FA, mean (s.d.)	0.16 (0.02)	0.16 (0.02)	0.16 (0.02)
			
*Mother characteristics*			
26-week EPDS raw score, mean (s.d.)	7.79 (4.66)	7.26 (4.30)	8.28 (4.93)
3-month EPDS raw score, mean (s.d.)	6.50 (5.14)	6.10 (4.85)	6.84 (5.38)
12-month BDI raw score, mean (s.d.)	6.93 (7.63)	6.18 (6.70)	7.63 (8.39)
24-month BDI raw score, mean (s.d.)	7.62 (7.47)	7.18 (6.99)	8.06 (7.96)
36-month BDI raw score, mean (s.d.)	7.48 (7.34)	6.53 (6.12)	8.39 (8.28)
54-month BDI raw score, mean (s.d.)	6.28 (8.19)	5.40 (6.63)	7.08 (9.38)
Prenatal maternal depression standardized score, mean (s.d.)	0.07 (1.03)	−0.05 (0.95)	0.18 (1.09)
Average postnatal maternal depression standardized score, mean (s.d.)	0.04 (0.91)	−0.03 (0.79)	0.10 (1.01)
Maternal ethnicity, %			
Chinese	52.3	49.6	54.9
Malay	30.2	34.5	26.2
Indian	17.4	15.9	18.9
Maternal education, %			
Primary school	5.2	5.4	5.0
Secondary school	29.2	26.8	31.4
Pre-university, diploma or technical course	37.8	37.5	38.0
University undergraduate level	24.9	27.7	22.3
Above university undergraduate level	3.0	2.7	3.3

Abbreviations: APGAR, Appearance, Pulse, Grimace, Activity, and Respiration; BDI, Beck's Depression Inventory; EPDS, Edinburgh Postnatal Depression Scale; FA, fractional anisotropy.

**Table 2:** Interaction effects, independent effects and main effects of prenatal and postnatal maternal depression on left and right amygdala volume in the overall, girls and boys samples

	*Volume*					
	*Left amygdala*			*Right amygdala*		
	*Overall*	*Girls*	*Boys*	*Overall*	*Girls*	*Boys*
*Interaction model*						
Interaction term	0.005	0.013	0.020	0.098	0.188	−0.017
						
*Reduced model*						
Prenatal depression	0.095	0.107	0.103	0.097	0.219*	−0.005
Postnatal depression	0.030	0.009	0.079	−0.003	−0.050	0.046
						
*Separate models*						
Prenatal depression	0.109	0.111	0.139	0.096	0.195*	0.015
Postnatal depression	0.074	0.055	0.125	0.042	0.044	0.043

Abbreviation: MRI, magnetic resonance imaging.

Standardized *β* values are listed in the table. Note, *P<0.05 level. Covariates adjusted for: age at MRI, total brain volume, maternal ethnicity and maternal education.

**Table 3:** Interaction effects, independent effects and main effects of prenatal and postnatal maternal depression on left and right amygdala FA values in the overall, girls and boys samples

	*FA values*					
	*Left amygdala*			*Right amygdala*		
	*Overall*	*Girls*	*Boys*	*Overall*	*Girls*	*Boys*
*Interaction model*						
Interaction term	−0.025	−0.146	0.031	−0.046	−0.213	0.057
						
*Reduced model*						
Prenatal depression	0.026	−0.018	0.038	−0.020	−0.053	−0.003
Postnatal depression	0.105	0.165	−0.016	0.233**	0.348**	0.042
						
*Separate models*						
Prenatal depression	0.079	0.063	0.030	0.097	0.116	0.019
Postnatal depression	0.117	0.157	0.004	0.223**	0.325**	0.041

Abbreviations: FA, fractional anisotropy; MRI, magnetic resonance imaging.

Standardized *β* values are listed in the table. Note, **P<0.01 level. Covariates adjusted for: age at MRI, maternal ethnicity and maternal education.
