# Supplementary material for: Influences of prenatal and postnatal maternal depression on amygdala volume and microstructure in young children
Source: Transl Psychiatry. 2017 Apr 25;7(4):e1103–. doi: 10.1038/tp.2017.74 (PMC5416711; doi:10.1038/tp.2017.74)
Supplement: Supplementary Information [file tp201774x1.docx]

**Supplementary Material**

**MRI Home Training Program**

The MRI recruitment was started at a 4-year home visit. The researcher briefed the MRI procedure and showed the environment where the MRI is conducted. In the video, Radiologist Dr. Marielle V. Fortier introduced the MRI suite at KK Women’s and Children’s hospital and safety related to MRI. If parents agreed on the consideration of MRI, the materials for the MRI home training program were further briefed to parents. These materials included 1) the coloring book telling a story of going to the hospital for MRI; 2) the modified statue game, that is, when children hear music they can dance, while they hear MRI noise, they need to keep still in order to be rewarded stickers; 3) the video showing what to do and not to do in the scanner. The reminder calls for the home training were made 2 weeks and 3 days prior to the MRI visit.

**MRI On-Site Training**

During the MRI visit, three training sessions were conducted. In the first session, parents and children first watched the same video to get familiar with MRI and MRI scanning procedure on what to do and not to do. Children are verbally instructed on why they have to undergo the MRI investigation and on the importance of minimizing motion. In the second session, children sit next to the mock scanner. They are told that they have to practice in the mock scanner to become familiar with the MRI environment. The various parts of the MRI unit are demonstrated (coil, bed, head coil, headphone), and each step of the MRI investigation is explained. Also, the various MRI sounds are played at increasing volumes for the children to become accustomed to. To familiarize children with these sounds, the trainer associated the sounds of the various scan sequences with familiar sounds, e.g. of a ship. For this part of the training, children can bring a toy (animal) that is actually placed in the scanner during instruction. In the last session, children are encouraged to lie down in the mock scanner, equipped with headphones, and immobilized with foam cushions. Parents maintain physical contact by touching children’s legs and are asked to verbally encourage children as much as possible during the training session. The trainer needs to instruct children for their behavior and give them feedback.

**Table S1.** Demographics of the sample with good MRI data (study sample) and the dropout sample due to MRI image quality (dropout sample).

| **Measure** | **Study Sample**  **(N=235)** | **Dropout sample**  **(N=77)** |
| --- | --- | --- |
| ***Child Characteristics*** |  |  |
| Gestational Age (week), mean (*SD*) | 38.75 (1.20) | 38.63 (1.65) |
| Birth weight (gram), mean (*SD*) | 3111.69 (412.38) | 3071.05 (436.88) |
| APGAR Score, mean (*SD*) | 9.00 (0.07) | 8.99 (0.12) |
| Gender, Male/Female | 113/122 | 45/32 |
| Age (year), mean (*SD*) | 4.58 (0.08) | 4.56 (0.07) |
| ***Mother Characteristics*** |  |  |
| Prenatal Maternal Depression standardized score, mean (*SD*)  Average Postnatal Maternal Depression standardized score, mean (*SD*) | 0.07 (1.03)  0.04 (0.91) | 0.10 (0.99)  0.02 (0.83) |
| Maternal Ethnicity, %  Chinese  Malay  Indian | 52.3  30.2  17.4 | 51.9  24.7  23.4 |
| Maternal Education, %  Primary School  Secondary School  Pre-university, Diploma or Technical course  University Undergraduate level  Above University Undergraduate level | 5.2  29.2  37.8  24.9  3.0 | 5.3  25.0  34.2  32.9  2.6 |


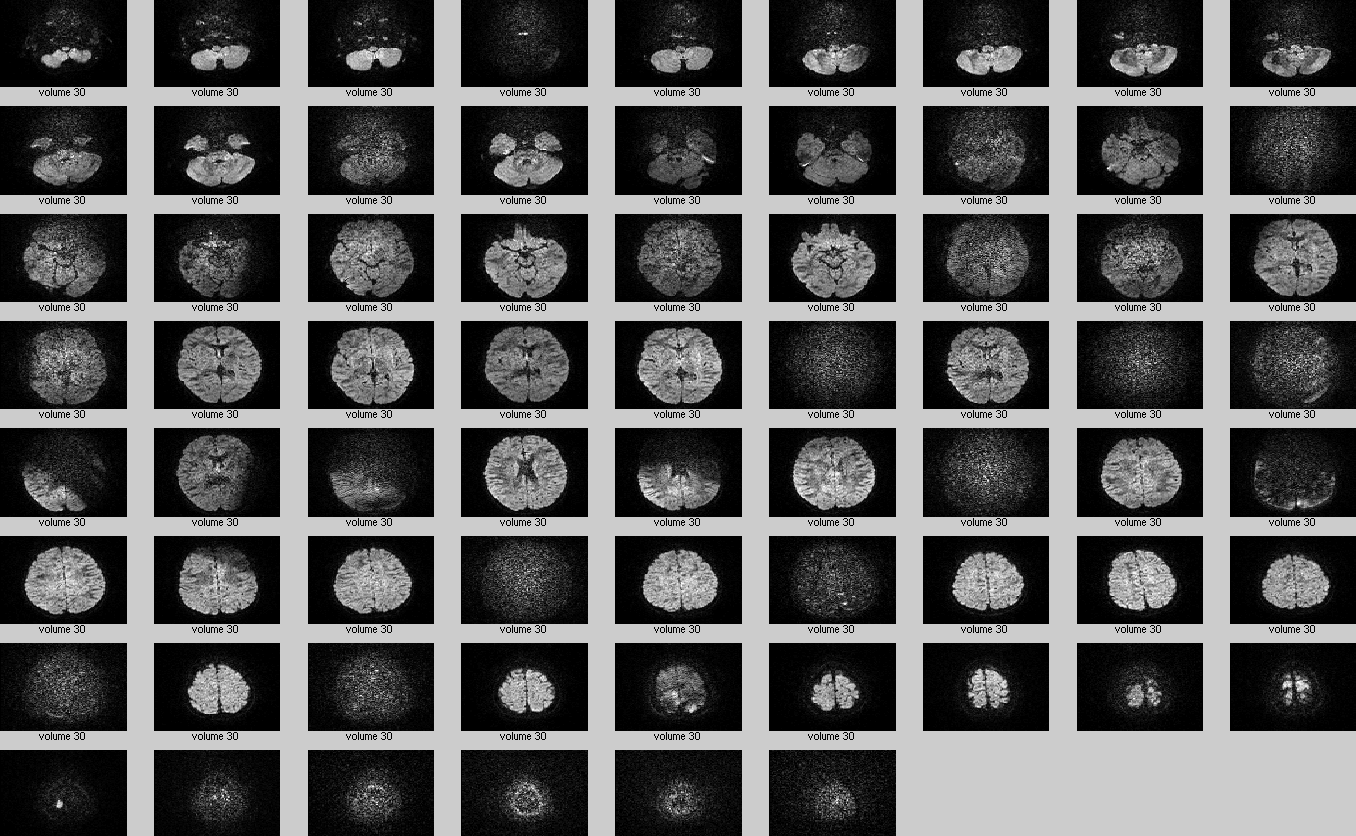


**Figure S1.** Example of diffusion weighted images with signal loss due to head motion.
